# The Neuroanatomical Correlates of Bladder Filling: An Activation Likelihood Estimation Meta-Analysis of Functional Neuroimaging Studies

**DOI:** 10.3390/neurolint17100156

**Published:** 2025-09-30

**Authors:** Christoph Müller, Albert Kaufmann

**Affiliations:** 1 Department of Internal Medicine, Lahn-Dill-Kliniken, 35578 Wetzlar, Germany; 2 Department of Internal Medicine, University of Marburg, 35037 Marburg, Germany; 3 Department of Neurourology, Swiss Paraplegic Center, 6207 Nottwil, Switzerland

**Keywords:** bladder filling, urine storage, voiding, activation likelihood estimation meta-analysis, functional neuroimaging, interoception

## Abstract

Background: Urinary continence relies on a complex interplay between urine storage and voiding involving both spinal reflex circuits and supraspinal brain areas to coordinate volun-tary control over emptying. Despite a vast number of studies on the pathophysiology of neurogenic bladder and urge incontinence, less is known about the central correlates of bladder filling. Methods: An ALE (activation likelihood estimation) meta-analysis including a total count of 14 studies investigating 243 participants under different conditions of bladder filling during functional neuroimaging was performed to demonstrate the neuroanatomical correlates of bladder filling. The literature search and reporting were conducted according to the PRISMA-P 2020 guideline. Data analysis was performed using the GingerAle software version 3.0.2 and was displayed with the Mango software 4.1 on an anatomical MNI template. Results: Synthesizing studies on the functional neuroanatomy of urine storage, bihemispheric clusters of activation in the thalamus, the insula and the cingulate were observed. Conclusion: The present ALE meta-analysis indicates that the supraspinal representation of urine storage involves areas of autonomous–homeostatic processing which allow for the perception of the usually unconscious inner state of bladder filling and enable postponing and voluntary voiding.

## 1. Introduction

Urinary continence relies on a complex interplay between peripheral vesicourethral reflexes and supraspinal efferents which allow for the volitional control of micturition. The dualism of urine storage and voiding is implemented by somatic and autonomic nerves coordinating striated and longitudinal urinary muscles to ena-ble voluntary withholding and urination [1]. Urinary incontinence affects around 10–20% of the general population with an inter-study range between 5 and 70%, an increasing prevalence with advanced age and an average male to female ratio of 1:2 [2,3,4,5]. Depending on the localization of lesions in the neuronal pathways of storage and micturition, a characteristic clinical presentation of overactivated or lost urinary muscle tone can be observed. Pathologies leading to neurogenic bladder can affect supraspinal brain areas or may involve parts of bladder-to-bladder reflex circuits of which at least 15 are reported [6]. These may result in disturbed signal transmission of the sympathetic (hypogastric nerve, T10-L2), parasympathetic (pelvic nerve, S2-S4), and somatic (pudendal nerve, S2-S4) nervous system projecting to and receiving input from nuclei in the lateral pons [7]. These coordinate four major urinary muscles, including the bladder neck, the bladder detrusor, and the internal and external urethral sphincters. The pontine micturition (PMC) and pontine storage center (PSC) receive visceroafferents from the bladder wall, integrate information on bladder filling or muscle tone, and send efferents back to the bladder and urethral muscles. Thereby a continuous monitoring of the filling state is achieved, which allows for a mostly unconscious adaptation of the vesicourethral muscles to maintain urinary continence.

These autonomic and somatic feedback loops can be disturbed on different neuroanatomical levels leading to five main types of urogenic bladder which can be categorized into (1) lower and (2) upper motor neuron dysfunction as well as (3) lesions above the PMC [8]. Dysfunctions of the sacral cord and sacral nerve root lead to low detrusor tone with intact internal urethral sphincter predisposing to overflow incontinence. A lower motor neuron mixed type A neurogenic bladder causes a flaccid detrusor with hypertonic external urinary sphincter, resulting in urinary retention. On the contrary, mixed type B neurogenic bladder, caused by lesions of the pudendal nucleus, is associated with a flaccid external urinary bladder and disinhibited detrusor nucleus, causing urinary incontinence [9]. An upper motor neuron bladder is characterized by spasticity of the bladder and sphincters, leading to detrusor–sphincter dyssynergia and predisposing to both ureteral reflux and incontinence. Among the different categories, supraspinal lesions above the PMC may be the most common cause of neurogenic bladder, which may result from cerebral ischemia, Parkinson’s disease, or multiple sclerosis [10,11,12].

The overall inhibitory effect of supraspinal brain areas on bladder function can be clinically observed in post-stroke patients who regularly present with urge incontinence related to detrusor overactivity. The conscious sensation of the bladder’s filling state correlates with activity in cerebral areas processing the homeostatic state of inner organs. These particularly include the insular and cingulate cortex, which are highly interconnected and provide the neuroanatomical substrate for the perception of autonomic disbalances [13]. Theoretically, the conscious sensation of usually unconscious inner states can be interpreted within the framework of interoceptive inference. It postulates that higher-order neuronal areas make predictions about an anticipated autonomic state which is continuously compared to visceroafferent feedback [14]. In bladder filling, a conscious perception may arise when a certain threshold is reached, diverging from homeostatic reference states which are most likely represent-ed in the insular cortex. An efferent response may be induced by the cingulate cortex and projections to other areas related to autonomic processing in the brainstem, mid-brain, and the hypothalamus [15]. The functional neuroanatomy of urine storage can be investigated with experiments of passive or active bladder filling during functional neuroimaging. Since voluntary con-trol over voiding seems to depend particularly on the conscious sensation of bladder filling, the present work focuses on the supraspinal representation of urine storage. For simplicity, the terms bladder filling and urine storage are used synonymously and refer to a vesical filling state of which the individual can be aware of. An activation likeli-hood estimation (ALE) meta-analysis of functional neuroimaging studies was con-ducted using the software GingerAle version 3.0.2 and displayed with the Mango software 4.1 [16,17,18].

## 2. Methods

**Figure 1:**
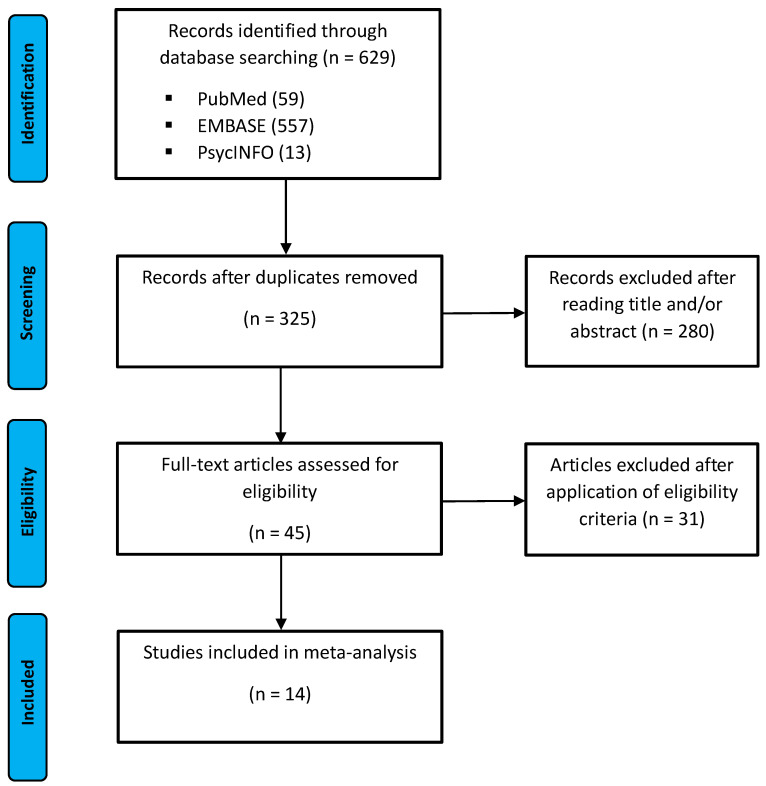
Flow chart of the study selection according to the Preferred Reporting Items for Systematic Reviews and Meta-Analyses (PRISMA).

The literature search was conducted and reported according to the Preferred Reporting Items for Systematic Reviews and Meta-Analyses (PRISMA-P 2020) guideline as illustrated in Figure 1 [19]. The PRISMA checklist can be found in the Supplementary Material and the study was registered on the open science framework (https://doi.org/10.17605/OSF.IO/4QJK2, first accessed on 10 September 2024).

### 2.1. Data Sources and Search Strategy

A comprehensive literature search was conducted across the databases PubMed, EMBASE and PsycNet until 30 May 2025. The search strategy included the combination of each term with Boolean operators and search by proximity. The keywords and Medical Subject Heading (MeSH) terms “bladder filling” OR “urine storage” AND “fMRI” OR “PET” OR “functional neuroimaging” in title and/or abstract were applied. No restrictions with regard to language, publication date, or type of article were implemented. All references were exported with the Citavi^©^ software (Lumivero, Denver, CO, USA) and duplicates were removed. The literature search was conducted with the following search string: (“bladder filling” [Title/Abstract] AND “fMRI” [Title/Abstract] OR “PET” [Title/Abstract] OR “functional neuroimaging” [Title/Abstract]), (“urine storage” [Title/Abstract] AND “fMRI” [Title/Abstract] OR “PET” [Title/Abstract] OR “functional neuroimaging” [Title/Abstract]).

### 2.2. Eligibility Criteria

The final meta-analysis only included prospective experimental/interventional studies using functional neuroanatomy to investigate bladder filling and urine storage under different experimental conditions. Articles were excluded if they were reviews, letters or comments, editorials, case reports or case series, and lacked information about the above-mentioned eligibility criteria or did not report foci coordinates. No restrictions considering publication date or language of published articles were applied.

### 2.3. Study Selection

The identified records were screened for eligibility by reading titles and abstracts of each article. If the eligibility criteria were met, the full text was obtained and investigated for inclusion in the meta-analysis. The x, y, z coordinates of all foci were extracted manually into a text file including the reference space, study name, condition, and sample size. All foci data were converted to the MNI (Montreal Neurological Imaging) coordinate space before entering the calculation of the ALE meta-analysis. The transformation into MNI space coordinates was conducted with the BioImageSuite online application (https://bioimagesuiteweb.github.io/webapp/mni2tal.html, accessed on 28 May 2025). The statistical analysis included studies investigating the functional neuroanatomy of urine storage.

### 2.4. Data Extraction and Statistical Analysis

The ALE meta-analysis was conducted using the software GingerALE version 3.0.2 (http://brainmap.org/ale/index.html, accessed on 18 July 2025) in an automated four step procedure. (1) At first, the foci data were entered into the GingerALE software which calculates the ALE values of each voxel using a 2 × 2 × 2 mm^3^ matrix. The ALE technique models the uncertainty of locations in a three-dimensional space as a Gaussian probability distribution centered about a peak at the reported coordinate. Using a random-effects model, a full-width half maximum (FWHM) depending on the subject size is applied to reflect the average smoothness of the input data. A wider FWHM is applied in data sets with a smaller subject size. Thereby a statistical map in which each voxel represents the probability that activation occurs is created. (2) The second step involves a permutation test to determine the statistical significance of each ALE value for which a random data set representing the null distribution at each voxel is calculated using a Monte Carlo simulation. The permutation test was run with 1000 permutations and an ALE map with a *p*-value for each voxel was determined. (3) A cluster-level inference was used to find clusters of data above a defined threshold. The ALE map was thresholded with a *p* < 0.05. (4) The cluster-forming threshold was *p* < 0.001 for a minimum cluster size of 100 mm^3^. The calculation of ALE maps was performed for all included studies to find the within-condition effect for the different conditions. The ALE maps were plotted on an anatomical MNI template using the Mango software version 4.1.

## 3. Results

### 3.1. Study Selection

The study selection process is illustrated in the flow chart in Figure 1. There was a total count of 629 records identified after the initial search including 59 from PubMed, 557 from EMBASE and 13 from PsycNet. After the removal of all duplicates, the remaining records were screened for eligibility. Finally, 14 studies were included in the meta-analysis. An overview on the characteristics of all included studies is provided in Table 1 and Table 2. Studies included were published between June 1998 until February 2021 and had a prospective observational/interventional study design. The applied imaging modality was fMRI in 10 studies [20,21,22,23,24,25,26,27,28,29], PET in 3 studies [30,31,32], and SPECT (single-photon emission computed tomography) in 1 study [33]. The functional neuroanatomy of urine storage was investigated by active filling of the bladder via urethral catheter in 9 studies and by passive filling during urine withholding in 5 studies. The filling state was assessed by the sensation of a filled bladder [21,25,26,27,28,30,31,32,33], intravesical volume [22,23,24,29], or intravesical pressure level [20].

In total, the final ALE meta-analysis included activity coordinates of 138 foci. Summarizing the sample characteristics of all included studies, a count of 243 healthy individuals with a mean age of 35.8 years, of which 148 (60.9%) were female, was investigated. Despite the presumed interstudy methodological variability of the functional neuroanatomy in urine storage, all studies exhibited patterns of activation in the insular and/or cingulate cortex [20,21,22,23,24,25,26,27,28,29,30,31,32,33].

### 3.2. ALE Cluster Analysis

**Figure 2:**
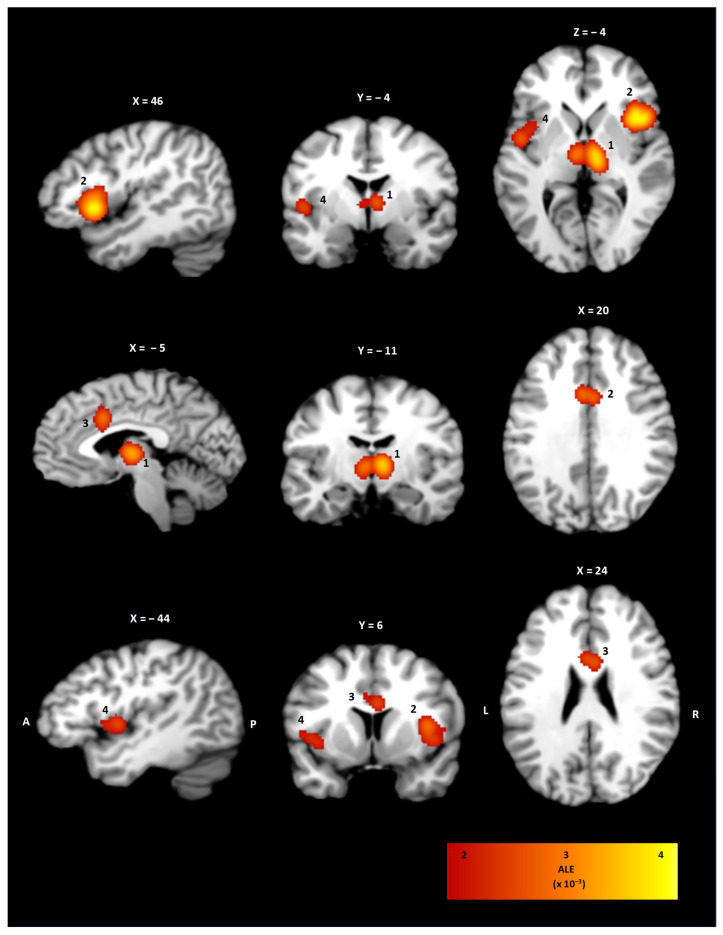
Activity clusters of the ALE meta-analysis in sagittal, coronal, and transversal view. Statistically significant activation was observed for the right and left thalamus (1), the right insula (2), the cingulate cortex (3), and the left insula (4). The ALE value of each voxel is color-coded according to the bar on the right.

The ALE meta-analysis of all included studies investigating the neuroanatomical correlates of urine storage resulted in four statistically significant clusters of activity as shown in Table 3 and Figure 2. On the right hemisphere, a cluster of 10,928 mm^3^ with coordinates for the weighted center in (44, 15, 4) involving the insula in Brodmann’s area (BA) 44 was observed. Correspondingly, the left insula showed a statistically significant activation with coordinates for a weighted center in (−44, 3, 3; BA 13). In addition, a bilateral thalamic cluster (9352 mm^3^, 6, −14, 4) was shown. In addition, statistically significant activation in the right and left cingulate cortex (3392 mm^3^, −1, 12, 29; 24) was observed.

## 4. Discussion

The presented ALE meta-analysis demonstrates the central neuroanatomical correlates of urine storage by summarizing studies investigating passive or active bladder filling using functional neuroimaging. Statistically significant activation of clusters was found in the thalamus, the insula, and the cingulate cortex bilaterally. The results substantiate the idea of central autonomous processing of visceroafferent information by the insular and cingulate cortex. Their activation may not only give rise to a conscious sensation from a certain threshold but may also induce an affective and autonomous response [34].

**Figure 3:**
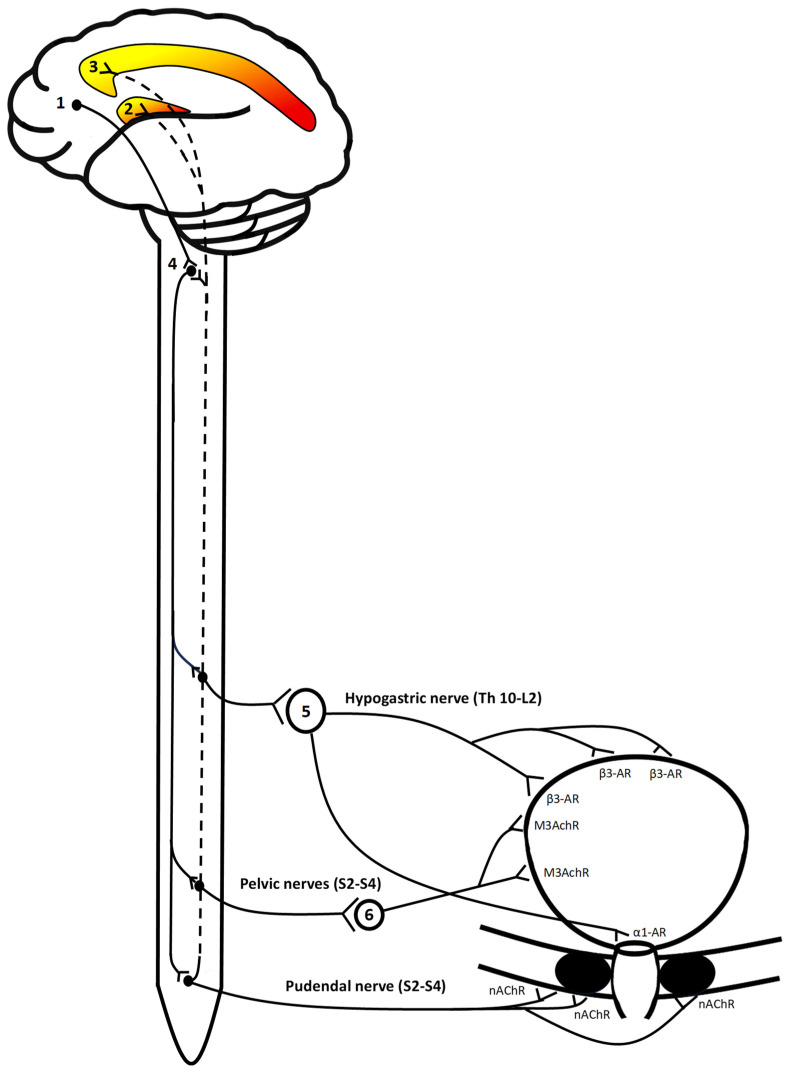
Simplified illustration of the neural control of urine storage. Central processing involves the (pre-) frontal cortex (1) receiving input from the cingulum (3), which is activated by the insular cortex (2) if a certain threshold of divergence from homeostatic set-points is reached. Activation of the prefrontal cortex (1) causes efferent signals to be sent to the dorsal pons (4), where the PMS and PSC are located. Descending signals from the PMC activate parasympathetic preganglionic sacral motor neurons synapsing in the major pelvic ganglion (6) with neurons of the pelvic nerves releasing acetylcholine on the bladder’s detrusor. Efferent signals from the PMC (4) activate GABAergic interneurons inhibiting preganglionic sympathetic neurons synapsing in the inferior mesenteric ganglion (5) that would activate alpha- and beta-adrenergic receptors in bladder wall and internal urethral sphincter. Somatic neurons in the pudendal nerve activate the external urethral sphincter by stimulating nicotinic acetylcholinic receptors. Efferents from the PSC (4) activate motoneurons in the sacral spinal cord, which cause contraction of the external urethral sphincter by norepinephrine release. Visceroafferent signals are conveyed within the hypogastric and pelvic nerves as indicated by the dashed line and either synapse with neurons in the PMC or are relayed from the thalamus or periaqueductal grey (not depicted) to central areas of homeostatic regulation (2, 3).

Sensory input related to the activation of neural structures representing the cen-tral correlates of bladder filling is transmitted by distinguishable visceroafferent path-ways. The sensory information from the bladder is encoded by low or high threshold mechanoreceptors and is conveyed via myelinated Aδ or unmyelinated C fibers which enter the spinal cord via the hypogastric and pelvic nerves [35]. Sensory information on the passive distension and active contraction of the bladder wall is conveyed via Aδ fibers stimulated by mechanoreceptors and C fibers sending input about non-mechanical, noxious stimuli within the bladder [36]. Visceroafferents from the bladder have their first-order neurons in the dorsal root ganglia (DRG), which project to Rexed layer V in the spinal cord and may then either synapse with neurons in the dorsal horn or the Ncl. gracilis/cuneatus in the brainstem [37]. Accordingly, visceroafferents ascend by either the dorsal system or the spinothalamic/spinotegmental tract and connect with thalamic nuclei from where projections are sent to cortical areas, e.g., the insular and cingulate cortex. While these projections allow for the conscious sensation of bladder filling, afferents to subcortical nuclei like the periaqueductal grey are essential for bladder-to-bladder reflexes to enable urinary continence [6]. Both the subconscious reflexes and the voluntary control of micturition depend on efferent signals from nuclei in the dorsal pons, i.e., the PSC and PMC [38]. Urine storage is implemented by efferents from the PSC activating motoneurons in Onuf’s nucleus, which release acetylcholine on the external urethral sphincter via the pudendal nerve [39]. In addition, sympathetic neurons in the lumbar spine are activated and connect with neurons in the inferior mesenteric ganglia, which then inhibit the bladder detrusor and induce contractions of the internal urethral sphincter by norepinephrine release. The switch to micturition is enabled by the PMC, which is activated by supraspinal neurons if a certain threshold of bladder filling is reached [40]. The PMC sends efferents to parasympathetic motoneurons in the sacral spinal cord which then synapse with postganglionic parasympathetic neurons causing detrusor contraction by acetylocholine release on muscarinic receptors. The activation of GABAergic spinal interneurons by the PMC inhibits motoneurons in Onuf’s nucleus, causing relaxation of the external urethral sphincter (Figure 3) [41].

Conceptually, the PMC seems to act as a switch that integrates both ascending afferents on the bladder’s filling state and descending motor commands to the vesicourethral muscles. A stronger stimulation by cortical areas causes the PMC to activate parasympathetic motor neurons and GABAergic interneurons in the spinal cord which facilitates voiding by detrusor contraction and sphincter relaxation [41]. Sensory information on the bladder’s filling state seems to be transmitted primarily via visceroafferents within the hypogastric and pelvic nerves, while somatoafferents of the pudendal nerve innervate the urethra and urogenital diaphragm [42]. Accordingly, information related to urine storage should be processed primarily by cortical areas involved in the homeostatic regulation of inner organs rather than by somatosensory brain regions. This assumption is in line with the observed bilateral activation of the insular and cingulate cortex. Viscerosensory and somatosensory information is conveyed via distinguishable spinal pathways connecting with thalamic nuclei from where projections are sent to areas of either somatosensory or autonomous processing. In general, viscerosensory information is transmitted by the phylogenetically older paleo- and archispinothalamic pathways which synapse with medial thalamic nuclei projecting to cerebral areas involved in the autonomous and affective–motivational aspects of sensory information [43,44]. Somatosensory input which is relayed via lateral thalamic nuclei projecting to the somatosensory cortex results in a sharp and easily localized sensation. Contrarily, the perception of visceral afferent stimuli is perceived as being more diffuse and poorly localized. Most of the visceroafferent input may remain subconscious while serving basic homeostatic needs and may only rise to consciousness if a certain threshold of divergence from an anticipated homeostatic state is reached [45].

The awareness of usually unconscious visceral stimuli is referred to as interoception and is implemented anatomically by visceroafferents projecting to the insular and anterior cingulate cortex (ACC) [46]. It is hypothesized that the insula provides homeostatic reference states against which the ascending sensory input is compared. Its activation would then result from a di-vergence or mismatch from these homeostatic states, which would lead to afferent signals being sent to the ACC. While the insula gives rise to the feeling of a disbalance of physiological states, the ACC attributes a motivational value, thereby creating basic emotions which drive future behavior towards an anticipated condition of homeosta-sis [47,48 ].

Anatomically, the insula is divided by the central sulcus into an anterior and posterior part. Stimulation of the anterior insula has been shown to evoke viscerosensory perceptions, whereas induced activation of the posterior insula is associated with somatosensory symptoms [49]. Accordingly, the anterior agranular cortex of the insula is connected to the limbic, the orbitofrontal, and the cingulate cortex, while the posterior part projects to the amygdala and the dorsal thalamus. Functionally, the insula can be divided into four regions, which integrate incoming information from posterior to mid-anterior [50]. Anterior–ventral areas of the insula show connections with the ACC and provide a socio-emotional value to visceral perceptions. While central parts of the insular cortex are related to olfactogustatory perceptions, the mid-posterior insula integrates incoming visual and auditory information, which may induce an adaptive motor response.

The cingulate cortex can be functionally divided into four posterior to mid-anterior areas. The anterior part, as part of the ventral or “what” stream, is involved in emotional salience and integration of incoming visceral sensations [51]. By processing autonomic states and sending it to the effector system, it may contribute to an adaptive response [52]. The medial cingulate cortex, which is connected to the posterior cingulate cortex (PCC), is engaged in behavioral decisions and is related to visuospatial orientation. The retrosplenial cortex contributes to the consolidation of memories by sending input to hippocampal areas [53]. As described earlier, the awareness of usually unconscious states is referred to as interoception and can be embedded within the concepts of predictive coding and active inference [54,55]. Accordingly, visceromotor areas (VMA), like the ACC, generate predictions about future sensory events which are compared with the actual sensory feedback. A mismatch between these predictions and the feedback from visceral afferents causes a prediction error which results in the consciously perceived sensation. This is structurally organized within visceromotor efferents, not only sending motor commands to effectors of inner organs but providing an efference copy for viscerosensory cortical areas [56]. In case of a mismatch, predictions generated by VMA are updated to match the actual sensory feedback. This process of neuronal adaptation, referred to as predictive coding, could be related to the activation of both the insular and cingulate cortex, which provide homeostatic reference states against which the feedback of visceral afferents is compared [57]. In addition to the emergence of a painful sensation, the prediction error induces a response of the effector system to correct for the detected disbalance. This control and regulation system includes an autonomic reflex circuit for the autonomic pathway and projections to higher motor areas for the somatosensory system. Theoretically, the induction of a behavioral response is related to the framework of ac-tive inference postulating that prediction errors induce the effector system to correct the sensory mismatch. This is implemented with the ACC being involved in response selection by sending efferents to higher-order motor areas [58]. Therefore, interoception as the perception of usually unconscious inner states can be viewed as a basic consciousness which guides the individual’s behavior towards regaining autonomic homeostasis.

During the conscious sensation of bladder filling, the individual is prompted to gain voluntary control over urine storage and find an acceptable situation to void. The crucial role of the insula and ACC in urine continence is demonstrated in the pathophysiological context of urge incontinence. Several studies have shown stronger activation of the insular and cingulate cortex in patients with urge incontinence compared to healthy controls during urine storage [59]. This observation can be reversed if physical treatment leads to improvement of urinary continence [60]. The influence of the insular and cingulate cortex on the volitional control of micturition is further substantiated by clinical findings indicating that cerebral damage in these areas is associated with an increasing incidence of urinary incontinence [61]. The observed stronger activation of the right insula is consistent with lesion studies in urge incontinence indicating that the right hemisphere seems to be more involved in the control of urine storage and voiding [62,63,64]. Neurophysiological studies also indicate functional lateralization of the insula in the autonomic control of homeostatic processes [65].

Symptoms of urge incontinence are associated with detrusor overactivity following disinhibition of the PMC. The loss of voluntary control over voiding is oftentimes related to dysfunctions of the prefrontal cortex which may result in enhanced activation of the PMC, causing stronger stimulation of the detrusor muscle. The regularly observed stronger recruitment of the insular and cingulate cortex in urge incontinence may be an attempt to compensate for the reduced activation of the prefrontal cortex [61]. A stronger compensatory activation of the insular cortex may lead to the feeling of ur-gency and the desire to void. While the insula integrates viscerosensory information of bladder filling, the activation of (pre-) frontal areas allow to postpone micturition and find a situation convenient to void. The inability of the prefrontal cortex to secure bladder filling and micturition may instead result in a stronger reliance on autono-mous–homeostatic processing by the insula and cingulum. This may explain the affec-tive dimension of urge incontinence, which is oftentimes described as a “sudden com-pelling desire to void that is difficult to defer” and is accompanied by a fear of leakage [8,66]. In addition, clinical evidence for the importance of the insular and cingulate cortex in regulating urinary continence is provided by lesion studies showing that cerebral in-farction in areas of homeostatic regulation may predispose to urinary incontinence [67,68]. Treatment-wise, the observed activation of the insular and cingulate cortex may imply a role for non-pharmacological treatment options which act by modifying central autonomous dysregulations. Sacral neurostimulation is commonly used in overactive bladder and its successful application is associated with reduced activation of the insula and ACC during symptoms of urgency [69]. Biofeedback seems to be particularly useful in the treatment of urge incontinence, as it may help in regaining voluntary control over pelvic floor muscles [70]. Although clinical trials are limited, the application of repetitive transcranial magnetic stimulation could help to alleviate symptoms in patients with neurogenic bladder dysfunction [71]. Conceptually, the observed activation of the insular cortex which receives visceroafferents from medial thalamic nuclei may alert the individual of ongoing homeostatic disbalance. With increasing divergence between the predicted and the actual homeostatic state, the sensation rises to consciousness and induces an anticipatory response.

Although the involvement of homeostatic supraspinal brain areas is a consistent finding across the included studies, some inter-study heterogeneity with regard to the study population and methodology should be assumed. Most included studies investi-gated younger healthy individuals with a female predominance, which may reduce the clinical generalizability of the results. Epidemiological studies indicate that both age and gender have a significant impact on the occurrence of urinary incontinence [2,3,4,5]. However, the observed activity pattern of the presented ALE meta-analysis contributes to an improved understanding of the voluntary control over micturition, which might be of particular interest in the context of urge incontinence and neurogenic bladder. The average sample size of 17 participants implies low statistical power for each individual study and argues for research synthesis despite the reported interstudy heterogeneity. In addition, the applied experimental set-up, including active or passive bladder filling and the defined threshold of the vesical filling state, might also be related to methodological heterogeneity. Study designs using urinary catheters might be associated with procedural discomfort and shorter filling times compared to studies with passive bladder filling. Moreover, the applied imaging modalities are associated with different temporal and spatial resolutions. In general, fMRI has a higher temporal and spatial resolution than PET. While fMRI allows for the investigation of ongoing neuronal activity, PET is associated with a time delay of at least 30 min between the injection of a radioactive tracer and measurable neuronal activity [72]. In SPECT, a radiopharmaceutical is stored intracerebrally shortly after injection and can be detected after 1–2 h, thereby capturing the preceding neuronal activity [73]. Despite the significant heterogeneity in temporal resolution, each study design accounts for the temporal delay of the applied imaging modality by its experimental set-up. Therefore, it appears methodologically reasonable to combine the activity coordinates of different imaging modalities. Given the restriction to a healthy study population, the observed findings may be of limited clinical generalizability. Future research may therefore focus on pathological aspects of bladder filling and investigate how treatment of urinary incontinence may alter the functional neuroanatomy.

## 5. Conclusions

The present ALE meta-analysis demonstrates that bladder filling leads to activa-tion of the insular and cingulate cortex which are related to the autonomous–homeostatic integration of visceroafferent input from inner organs. Their activation enables the individual to perceive the usually unconscious process of urine storage, gives it an affective–motivational dimension, and induces a conscious behavioral response. Theoretically, the perception of inner states can be explained within the framework of interoceptive inference. Future research may have a more clinical focus to get a better understanding of the pathophysiology of urge incontinence and neurogenic bladder by demonstrating their specific functional neuroanatomy.

## Figures and Tables

**Table 1:** Studies included in the meta-analysis.

Author (Year)	Study Design	Imaging Modality	Sample Size (% Female)	Mean Age ± SD (Years)	Overview on Conditions	Foci
Blok et al. (1998) [30]	Prospective experimental study	PET	18 (100%)	27.0 ± n.a.	15 min prior to micturition while withholding urine (scan 1), during micturition (scan 2), 15 min after micturition (scan 3), 30 min after micturition, contrasts between each condition	2
Dasgupta et al. (2005) [31]	Prospective experimental study	PET	8 (100%)	n.a.	Healthy participants and patients with urinary retention due to sphincter overactivity had 6 scans with empty bladder and full bladder; conditions with a sacral neurostimulator in patients with either empty or full bladder	3
Griffiths et al. (2005) [20]	Prospective experimental study	3 T fMRI	12 (92%)	n.a.	Trials with empty bladder and after infusion with normal saline at a rate of 60 mL/min via urine catheter; intravesical pressure was monitored	10
Zhang et al. (2005) [21]	Prospective experimental study	3 T fMRI	12 (0%)	23.8 ± 0.65	Conditions of empty and full bladder with either relaxed or contracted pelvic floor muscles; participants were instructed to drink until a feeling of bladder fullness was perceived, instruction to voluntarily contract the pelvic floor under both empty and full bladder conditions	10
Yin et al. (2006) [33]	Prospective experimental study	SPECT	15 (0%)	32.7 ± 7.3	Each participant was scanned with an empty bladder or during withholding urine with a full bladder	4
Mehnert et al. (2008) [22]	Prospective experimental study	3 T fMRI	8 (100%)	24.3 ± n.a.	Conditions of either empty or filled bladder by saline infusion via urethral catheter and an alternating resting period repeated five times, intermittent genital nerve stimulation	17
Griffiths et al. (2009) [23]	Prospective experimental study	3 T fMRI	10 (100%)	n.a.	CConditions of bladder filling via urethral catheter by infusion and partial withdrawal of bladder volume with up to 6 cycles	6

Abbreviations. fMRI, functional magnetic resonance imaging; n.a, not available; PET, positron emission tomography; SPECT, single-photon emission computed tomography, T, Tesla.

**Table 2:** Studies included in the meta-analysis.

Study ID			Population		Intervention	
Author (Year)	Study Design	Imaging Modality	Sample Size (% Female)	Mean Age ± SD (Years)	Overview on Conditions	Foci
Mehnert et al. (2011) [32]	Prospective experimental study	PET	14 (100%)	24.8 ± n.a.	Infusion of maximally 100 mL cold normal saline via urethral catheter, conditions of empty bladder, sensation of filled bladder, two conditions of continuous draining for 10 cycles	6
Nardos et al. (2014) [24]	Prospective experimental study	3 T fMRI	20 (100%)	56.2 ± n.a.	Infusion of normal saline via transurethral catheter at an infusion rate of 50 mL/min until urge to void, phase of withdrawal, trials with empty and filled bladder	3
Kruht et al. (2014) [25]	Prospective experimental study	3 T fMRI	23 (100%)	n.a.	Infusion of normal saline into the bladder at a rate of 50 mL/min, conditions of empty bladder, first sensation and strong desire to void, first filling with 100 mL normal saline and then rapid filling with additional 25 mL and withdrawal, two trials of filling and emptying	8
Gao et al. (2015) [26]	Prospective experimental study	3 T fMRI	30 (73%)	29.8 ± 5.9	Conditions of empty bladder after voiding and strong desire to void followed by phase of drinking water, evaluation of the desire to void on a VAS	11
Leitner et al. (2017) [27]	prospective experimental study	3 T fMRI	33 (48%)	35.3 ± 11.0	8 blocks consisting of infusion of 100 mL normal saline, plateau of perceived filling, rating of desire to void, short rest period, withdrawal of 100 mL normal saline, plateau phase after 100 mL normal saline have been withdrawn, rating of desire to void and pain level	27
Walter et al. (2019) [28]	Prospective experimental study	3 T fMRI	20 (50%)	39.2 ± 11.6	8 blocks of normal saline infusion via transurethral catheter, plateau phase of filled bladder, rating of desire to void, rest condition, withdrawal of 100 mL normal saline, plateau phase of filled bladder, rating of desire to void, resting condition, the first measurement was followed by a second scan after 5–8 weeks and follow-up interview	23
Pang et al. (2021) [29]	Prospective experimental study	3 T fMRI	20 (50%)	51.5 ± 5.6	2 resting state trials with an empty bladder and a full bladder; between both resting state scans, the bladder was filled with 200 mL of normal saline via urethral catheter, repeated infusion and withdrawal	8

Abbreviations. fMRI, functional magnetic resonance imaging; n.a.; not available; PET, positron emission tomography; T, Tesla; VAS, visual analogue scale.

**Table 3:** Location of clusters with statistically significant brain activation and coordinates for the weighted center for all included studies.

Cluster	Brain Region	BA	x	y	z	Volume (mm^3^)	ALE (×10^−3^)
1	Right/Left Thalamus	-	6	−14	4	9352	3.5
2	Right Insula	44	41	15	4	10,928	3.7
3	Right/Left Cingulate Cortex	24	−1	12	29	3392	2.7
4	Left Insula	13	−44	3	3	2912	2.5

Abbreviations. ALE, activation likelihood estimation; BA, Brodmann area.

## Data Availability

No new data were created or analyzed in this study.

## Supplementary Materials

The following supporting information can be downloaded at: https://www.mdpi.com/article/10.3390/neurolint17100156/s1.

## Abbreviations

ACC	Anterior cingulate cortex
ALE	Activation likelihood estimation
BA	Brodmann area
DRG	Dorsal root ganglia
fMRI	Functional magnetic resonance imaging
FWHM	Full width half maximum
GABA	Gamma aminobutyric acid
MeSH	Medical Subject Heading
MNI	Montreal Neurological Imaging
n.a.	Not available
PCC	Posterior cingulate cortex
PMC	Pontine micturition center
PRISMA	Preferred Reporting Items for Systematic Reviews and Meta-Analyses
PSC	Pontine storage center
PET	Positron emission tomography
SPECT	Single-photon emission computed tomography
T	Tesla
VAS	Visual Analogue Scale
VMA	Visceromotor areas
